# Imaging of peripheral vascular malformations — current concepts and future perspectives

**DOI:** 10.1186/s40348-021-00132-w

**Published:** 2021-12-07

**Authors:** Vanessa F. Schmidt, Max Masthoff, Michael Czihal, Beatrix Cucuruz, Beate Häberle, Richard Brill, Walter A. Wohlgemuth, Moritz Wildgruber

**Affiliations:** 1 Department of Radiology, University Hospital, LMU Munich, Munich, Germany; 2 Clinic for Radiology, University Hospital Muenster, Muenster, Germany; 3 Angiology Division, Department for Medicine IV, University Hospital, LMU Munich, Munich, Germany; 4 Clinic and Policlinic of Radiology, Martin-Luther University Halle-Wittenberg, Halle (Saale), Germany; 5 Department for Pediatric Surgery, Dr. von Haunersches Kinderspital, University Hospital, LMU Munich, Munich, Germany

**Keywords:** Vascular malformations, Duplex ultrasound, Magnetic resonance imaging, Molecular imaging, Positron emission tomography, Multispectral optoacoustic tomography, Thermography

## Abstract

Vascular Malformations belong to the spectrum of orphan diseases and can involve all segments of the vascular tree: arteries, capillaries, and veins, and similarly the lymphatic vasculature. The classification according to the International Society for the Study of Vascular Anomalies (ISSVA) is of major importance to guide proper treatment. Imaging plays a crucial role to classify vascular malformations according to their dominant vessel type, anatomical extension, and flow pattern. Several imaging concepts including color-coded Duplex ultrasound/contrast-enhanced ultrasound (CDUS/CEUS), 4D computed tomography angiography (CTA), magnetic resonance imaging (MRI) including dynamic contrast-enhanced MR-angiography (DCE-MRA), and conventional arterial and venous angiography are established in the current clinical routine. Besides the very heterogenous phenotypes of vascular malformations, molecular and genetic profiling has recently offered an advanced understanding of the pathogenesis and progression of these lesions. As distinct molecular subtypes may be suitable for targeted therapies, capturing certain patterns by means of molecular imaging could enhance non-invasive diagnostics of vascular malformations. This review provides an overview of subtype-specific imaging and established imaging modalities, as well as future perspectives of novel functional and molecular imaging approaches. We highlight recent pioneering imaging studies including thermography, positron emission tomography (PET), and multispectral optoacoustic tomography (MSOT), which have successfully targeted specific biomarkers of vascular malformations.

## Background

Vascular anomalies (VAs) comprise a wide spectrum of vascular lesions, which can involve any part of the body, including vascular tumors with altered cellular proliferation, and vascular malformations with underlying mesenchymal and angiogenetic dysplasia [1, 2]. Whereas infantile hemangiomas as the most frequent vascular tumor regularly regress with patient’s age, vascular malformations never regress on their own, but frequently increase in size and grow concomitantly or even overproportioned with the child. The most accepted classification of VAs, grounded in biological, histopathological, hemodynamic, and clinical findings, is provided by the International Society for the Study of Vascular Anomalies (ISSVA) [3–5]. The ISSVA classification was last updated in 2018 based on novel descriptions of genes involved in the development of VAs [6–10]. Congenital vascular malformations are divided into simple malformations, combined malformations, malformations of major named vessels, and malformations associated with other anomalies (syndromic vascular malformations) [11]. Due to the huge variety in clinical presentation depending on the lesion size, localization, involved tissue, and the subtype of the malformation these vascular pathologies present as a diagnostic challenge [2, 12]. Thus, besides the detailed clinical history and physical examinations, imaging plays a crucial role in the correct diagnosis and classification of vascular malformations. Imaging should be able to assess the exact flow dynamics in order to differentiate the various types of vascular malformations (fast-flow vs. slow-flow lesions), as to date there is a high rate of misdiagnosis [2]. In addition, the anatomical extent with tissue involvement and affected compartments should be precisely demonstrated for an interdisciplinary and multimodal treatment planning [13]. We performed a PubMed search of reports focusing on imaging of extracranial vascular malformations using the search terms “Imaging,” “Vascular malformations” and “Vascular anomalies” with a focus on recently published work including new treatment strategies. This review provides an overview of subtype-specific imaging requirements, current concepts, and established imaging modalities for the accurate diagnosis of vascular malformations. Beyond depicting the underlying phenotype, increasing knowledge of molecular profiles of vascular malformations and new treatment strategies make advanced imaging strategies mandatory. Thus, additionally, this review presents an outlook on novel molecular and functional imaging targeting specific features of these rare diseases.

## Major tasks of diagnostic imaging of peripheral vascular malformations

In most cases, the proper diagnosis of vascular malformations can be made upon clinical examination and additional ultrasound imaging. Additional cross-sectional imaging has to report the proper location, size, and extent of the VA as well as to clearly differentiate vascular malformations from vascular tumors [14]. In certain cases of syndromic vascular malformations, imaging has to screen for additional associated abnormalities [2]. Distinct evaluation of the flow characteristics helps to categorize the lesion according to the ISSVA classification. Finally, imaging is required for proper treatment planning by depicting the anatomy of the inflowing feeders, nidus, and draining vessels of the malformation which can be used to access the lesion as well as for treatment monitoring [15].

## Current imaging concepts

In the following section, established imaging modalities and their adequacy in the diagnosis of vascular malformations are reviewed. The diagnostic value of each modality for the different types of vascular malformation with regard to the flow characteristics is summarized in Table 1.

**Table 1:** Characteristic imaging features of vascular malformations

*Malformation type*	*All imaging modalities (diagnostic value in flow-characteristics)*	*MRI features*	*Ultrasound*
Slow-flow VM	Ultrasound (3) CDUS/CEUS (1/3) X-ray (1) MRI and MRV (3) Transvenous phlebography (3)	Septated lobulated mass without mass effect Phleboliths (low SI), fluid-fluid levels, low SI on T1WI, high SI on T2WI No flow voids on SE images Infiltrates tissue planes and possible surrounding oedema No arterial or early venous enhancement Slow gradual enhancement and diffuse enhancement on delayed images with some late enhancement (≥ 5 s) Normal afferent arteries Contrast pooling in dilated stagnant venous spaces in later venous phase imaging	Variable appearance (spongiform, multicystic, uncommon as dilated tubular channels) Usually heterogeneous, mostly hypoechoic intralesional phleboliths (calcifications) may be present Localized or involving multiple soft-tissue planes Spontaneous echo contrast, absence of spontaneous flow, luminal filling, and venous flow pattern detectable with provocative maneuvers (proximal or distal compression)
Slow-flow LM	Ultrasound (3) MRI (2)	Septated lobulated mass with some fluid–fluid levels Infiltration of tissue planes Low SI on T1WI, high SI on T2WI No flow voids on SE images Rim and septal enhancement No significant or slight diffuse enhancement	Macrocystic: Multicystic with thin septa Variable echogenicity of cystic contents, sometimes with fluid-fluid levels, especially if complicated with hemorrhage No flow or low vascular density on color Doppler, confined to septa Microcystic: Ill-defined and hyperechoic, containing scattered cysts < 1–2 cm Sometimes no visible cysts No flow or low vascular density on color Doppler
High-flow AVM	Ultrasound (1) CDUS/CEUS (3/3) MRI and MRA (2) Perfusion CT with CTA (3) Catheter angiography (3)	No well-defined mass Infiltration of tissue planes Flow voids on SE images Enlarged feeding arteries and draining veins Hypertrophied tortuous afferent arteries and efferent veins Direct arteriovenous communications via vascular nidus Early enhancement of arteriovenous lesion (≤ 5 s) Early enhancement of enlarged feeding arteries and nidus with shunting to draining veins	Conglomerate of tortuous, dilated vessels without discrete mass that can involve multiple soft-tissue planes Pulsatile flow with high velocities, low resistance, and spectral broadening

Diagnostic value: 1, moderate; 2, good; 3, high

### Ultrasound

Ultrasound represents the first imaging exam for patients with vascular malformations. By using ultrasound devices with the capabilities of color-coded Duplex ultrasound (CDUS) and pulsed wave Doppler (PW-Doppler), it is possible to assess both morphological characteristics as well as hemodynamic features (spectral waveform analysis, SWA) in real time [16, 17]. Additionally, contrast-enhanced ultrasound (CEUS) allows dynamic analysis of the microcirculation and perfusion [18] and might assist image-guided treatment, while distinguishing between sclerotized venous malformation (VMs) and untreated malformation portions [19]. Echocardiography may unreveal cardiac structural damage (e.g., valve incompetencies, ventricular dilatation) secondary to high-flow AVM/AVF.

Regularly, broad band high-resolution ultrasound (HR-US) probes with a frequency of at least 9–14 MHz are required for vascular lesions of the superficial soft tissue (2–30 mm). For deeper (> 30 mm) or intermuscular malformations and in case of obesity, 5–7 MHz probes are needed. Extremely small and superficial lesions, such as along the distal extremities, finger, or toe, higher frequency ranges (up to 24 MHz) are useful to maintain an accurate axial resolution. The water-bath method may be useful to overcome limitations in superficial lesions due to the small field of view, relative compressibility of the soft-tissue structures by the probe, patient motion, and/or discomfort from contact of the probe, particularly in children [20, 21].

Using brightness mode (B-mode), the morphological characteristics, including the exact extension of a lesion in two perpendicular planes, and the echotexture (hypo-, iso-, hyperechogeneities, homogeneous versus heterogenous signal) can be assessed. Furthermore, the relation of the malformation to the surrounding tissues can be evaluated, in order to reveal solid, “real” tumorous and proliferative parts, which may be found in differential diagnoses as hemangiomas [16]. Characteristic compressibility and pulsation during the examination may help to further classify the lesions. The clear identification of phleboliths presenting as hyperechogenic spots and posterior acoustic shadowing is a further advantage for lesion differentiation.

Using CDUS, vascular malformations can be categorized according to their flow characteristics. The precise type of vessels and associated fistulas within the lesion should be assessed. In case of slow intralesional blood flow, the pulse repetition frequency (PRF) needs to be gradually reduced while the color gain should be carefully increased without inducing blooming artifacts (blood flow signal extending beyond the vessel wall). The wall filter has to be turned off, and it is important to avoid the misinterpretation of movement artifacts as flow. It is crucial to be aware, that the “compression test” procedure may provoke a pendular flow even in stagnant malformations, which show absent flow signal without compression. Thus, a “polycystic” lesion, if repetitively compressed by the HR-US probe, may present with a typical color-shifting signal within the malformation [22].

The SWA is used to evaluate the intrinsic flow patterns, particularly in high-flow malformations: here, the distribution of flow velocities and typical spectral characteristics such as high diastolic flow (with a resistance index lower than 0.5) in shunt feeders can be demonstrated. In this case, altered signals in the veins may be indicative of the entity such as arteriovenous fistulas (AVFs) and arteriovenous malformations (AVMs) [16]. The shunt volume of such lesions can be estimated by integrating time-averaged flow velocity and vessel lumen cross-sectional area of the inflow arteries. CEUS, performed after the injection of strictly intravascular contrast agents by using contrast harmonic imaging, with a low mechanical index [23], is in combination with perfusion analysis an accurate tool to quantitative and dynamically assess the microcirculation of vascular malformations [24]. In addition, CEUS can be used for differentiation between the variant malformation types as the percentage of peak enhancement and the area under the curve are decreased in fast-flow malformations while the mean transit time is shorter in slow-flow malformations [24].

Besides diagnostic imaging, ultrasound is the imaging modality of choice for needle guidance during percutaneous sclerotherapy, embolization, or US-guided biopsies. Furthermore, ultrasound can be successfully applied for assessment of the therapeutic success by monitoring the degree of devascularization [25, 26]. Here, CEUS can quantify perfusion changes by using time–intensity curve analysis in digitally stored cine sequences [19].

A disadvantage of ultrasound imaging is the lack of precision regarding malformations located in deep tissues, adjacent to the bone or lung and abdomen or with regard to the involvement of adjacent critical structures such as the larynx/ pharynx or subfascial tissue and organs. Here, examination may not be effective for detailed microvascular imaging due to artifacts. In addition, US depends on the experience of the investigator, as respective parameters such as PRF and color-gain have to be adjusted accordingly.

### Magnetic resonance imaging

**Fig. 1:**
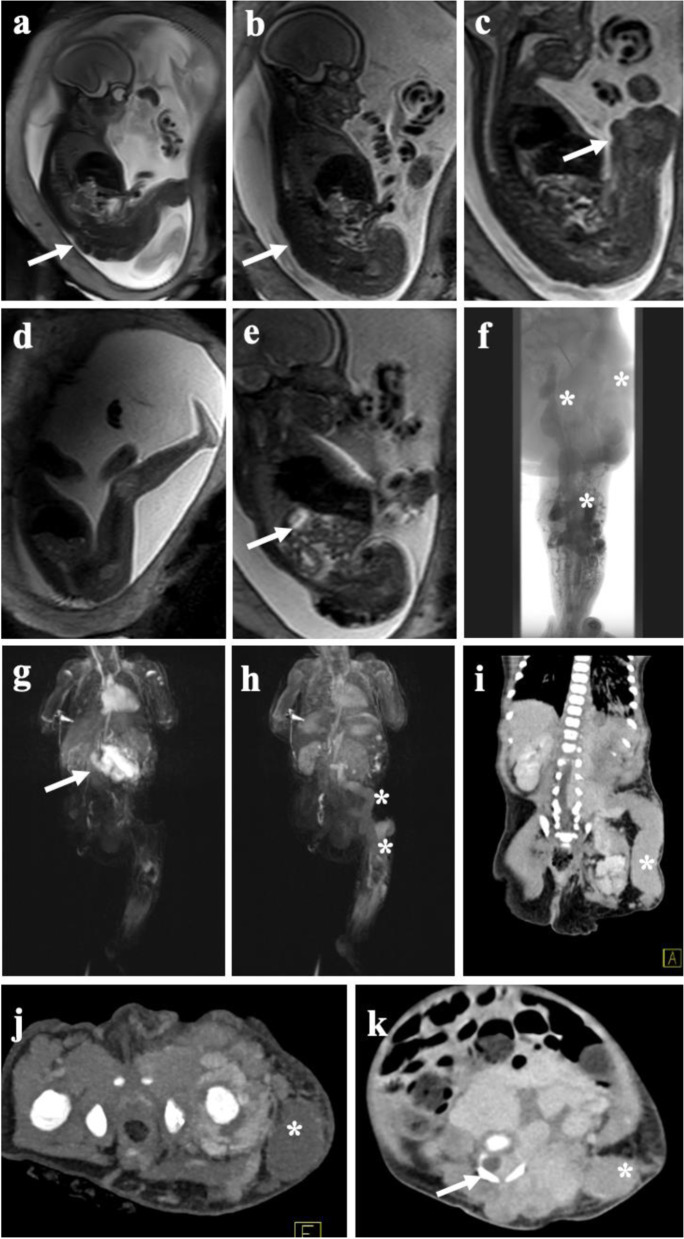
Fetal MRI and post-partum MRI/CT of complex vascular malformation. Fetal MRI using dedicated T2-weighted sequences reveal extensive venous malformation along the entire left leg (**a**) extending along the pelvis and lower back (**b**). **c** Large venous channel (arrow) raised suspicion of a large persistent marginal vein. **d** Healthy right leg in comparison. **e** Extension of the malformation in the retroperitoneal space. **f** Post-natal phlebography depicting large marginal vein together with extensive venous malformation of the lower and upper leg with extension into the pelvis (asterisk). Large blood volumes trapped in the marginal vein induced a localized consumptive coagulopathy in the child requiring immediate anticoagulation upon delivery. **g** Postnatal MRI revealing early arterial filling of an additional mesenteric arteriovenous malformation in the same patient, being asymptomatic at time of diagnosis. The course of the marginal vein, as depicted on MRI (**h**) and CT (**i**) showing large venous drainage into the pelvis (asterisk). **j** Transverse CT image in the arterial phase demonstrating multiple AV fistula paraspinal and along the left hip joint (arrows) together with the not yet contrasted marginal vein (asterisk). **k** Venous phase CT depicting the extension of intraabdominal, retroperitoneal, and paraspinal venous cavities with infiltration of the spinal canal (arrow) by the large slow-flow malformation, at time with contrast in the large marginal vein (asterisk). The large venous malformations required multiple rounds of image-guided embolization followed by stepwise surgery of the marginal vein

MRI is the most valuable diagnostic tool for comprehensive assessment of vascular malformations, as it combines high-contrast soft tissue imaging including tissue characterization, vascular imaging including flow measurements, and perfusion imaging. The major disadvantage especially in young children is the long examination time and susceptibility to motion artifacts, requiring sedation or anesthesia. There are no strict age limitations, however, MRI in children below 5 years usually has to be performed in deep sedation or general anesthesia. In selected cases prenatal MRI (Fig. 1) can help to assess complex malformations with potential impact on the child’s health immediately after birth as well as the mode of delivery including larger complications such as nonimmune hydrops fetalis and high-output cardiac failure secondary to fast-flow VA and arteriovenous shunting [27]. As prenatal MRI distinguishes the location, morphological components, and flow components of a lesion, it provides information allowing for distinct in utero differential diagnosis of tumors and vascular malformations [28, 29].

In general, MRI examination protocols classically consist of the subsequent sequences: spin echo (SE) or fast spin echo (FSE) T1w, T2w with fat saturation or short tau inversion recovery (STIR), fast 3D gradient-echo (GRE) T1w sequence, and dynamic contrast-enhanced MRA (DCE-MRA) in cases of suspected or confirmed fast-flow lesions [30, 31]. Fat suppression is particularly essential if the lesion involves the subcutaneous tissues as it increases the contrast between the malformation and the adjacent fat [32]. Due to the possibility of acquiring images in subsequent phases (early arterial, arterial, intermediate, and venous) as well as the high spatial and temporal resolution, dynamic contrast-enhanced imaging can separate arterial inflow from venous drainage as well as detect arterio-venous shunting. In addition, dynamic MRA allows to obtain information concerning the flow direction and contrast kinetics over time [31, 33].

Further MR angiographic techniques without intravenous contrast, such as time-of-flight (TOF) or phase contrast (PC) sequences, which do not expose the patient to contrast agents, are less used in the evaluation of VAs owing to their high susceptibility towards different flow velocities and associated flow-related artifacts [34].

Post-contrast MRI is useful to examine slow-flow malformations including the extent of their drainage in the venous system and characterization of mixed veno-lymphatic malformations [35].

### Computed tomography

Contrast-enhanced multi-slice CT allows for rapid assessment of vascular malformations with precise evaluation of the feeding and draining vessels, though the major disadvantage of this modality is the significant dose of ionizing radiation [36], which is particularly relevant in children [37]. Accordingly, CT is not recommended as a routine diagnostic modality but should be reserved for certain cases, where MRI is either not possible or expected to not provide enough information for proper treatment planning [13], which may allow to reduce procedure time and radiation dose during DSA [38]. This is ideally performed by time-resolved CTA and CT venography (4D CT imaging) over a wide *z*-axis coverage combined with low tube voltage settings [39]. Henzler et al. published an initial experience concerning 4D CTA in 7 pediatric patients with VMs considered valuable for treatment planning. However, in particular, the radiation dose of 4D CTA must be weighted in each case individually against the diagnostic performance in these often young patients [39].

### Arterial angiography and phlebography

Arterial arteriography and phlebography are imaging modalities immediately preceding a potential therapeutic procedure while in times of dynamic MR angiography they should not be performed as mere diagnostic tools anymore, owing to the invasive character and ionizing radiation exposure. Periprocedural invasive catheter angiography of fast-flow malformations provides selective assessment of arteriovenous shunting, nidus, and draining veins prior to embolization. Here, periprocedural angiography aims to understand specific flow dynamics, which frequently change in control angiography during embolization. In slow-flow malformations, direct percutaneous phlebography offers detailed information on intralesional flow patterns and potential connections to the draining deep venous system of the lesion before sclerotherapy [13]. Diagnostic arterial angiography is not indicated in slow-flow malformations, as they only contain hypodynamic small arteriovenous fistulas [40].

## Subtype-specific imaging findings

Based on their flow characteristics, the ISSVA further classifies simple vascular malformations comprising of one dysplastic vessel type into slow-flow lesions (92%), including capillary malformations (CMs, 10%—with respect to all malformations), venous malformations (VMs, 72%), and lymphatic malformations (LMs, 10%) as well as fast-flow lesions (8%), including arterio-venous malformations (AVMs) and arterio-venous fistula (AVFs) [5, 41]. Hereinafter, an overview of these subtypes and their specific imaging findings is provided covering morphologic and hemodynamic aspects.

### Slow-flow malformations

#### Capillary malformations

*CMs* are found in 0.1–2% of the population. As anomalies of the capillary networks within the skin and mucosal membranes, they can occur isolated or signalize the presence of a syndromic disease as Klippel–Trenaunay (KTS), Sturge–Weber (SWS), or Proteus [12]. The major groups of capillary malformations, according to the clinical patterns, are naevus simplex, naevus flammeus, cutaneous and/or mucosal CM (port-wine stains), reticulated capillary malformation (RCM), capillary malformation–arteriovenous malformation (CM-AVM), cutis marmorata telangiectatica congenita (CMTC), and telangiectasia [42–44]. Naevus simplex, the most frequent pattern of CMs, presents at birth with pale pink color and ill-defined borders while fading over time. Most frequent sites include mid forehead, upper eyelids, philtrum, and nape of the neck, followed by the occipital and parietal scalp, upper back, and the lumbosacral area [45]. Unlike the naevus simplex, the naevus flammeus, presenting as a pink or red patch on a newborn’s skin, may be located at any part of the body and persists throughout life [46]. If high-frequency ultrasound is performed (> 12 MHz), CMs present in form of hypoechoic very superficial lesions in depth from 0.2 to 3.7 mm showing increased vascularity on CDUS [47]. Using MRI, CMs can appear as a subtle signal abnormality within the subcutaneous fat tissue with surrounding skin thickening [48].

However, CMs can typically be diagnosed accurately by physical examination alone, and imaging is not needed for the diagnosis. In addition, there are rarely any specific imaging questions regarding isolated CMs, in particular as they regularly do not require endovascular therapies and thus no corresponding imaging treatment planning. Though, if port-wine stains (PWS) should be treated with pulsed dye laser (PDL), there are some reports regarding a noninvasive diagnostic technique called laser speckle contrast imaging (LSCI). LSCI, a relatively quick, non-contact, near-infrared-based imaging system providing a 2-dimensional image of perfusion maps of large biological surfaces, is descripted to be an efficient tool to measure perfusion reduction during PDL treatment sessions [49, 50]. In the case of CMs presenting associated with complex syndromes, here, MRI and CT are helpful tools to evaluate especially central nervous system involvement and to detect further manifestations as progressive gyral atrophy or cortical calcification [51].

#### Venous malformations

**Fig. 2:**
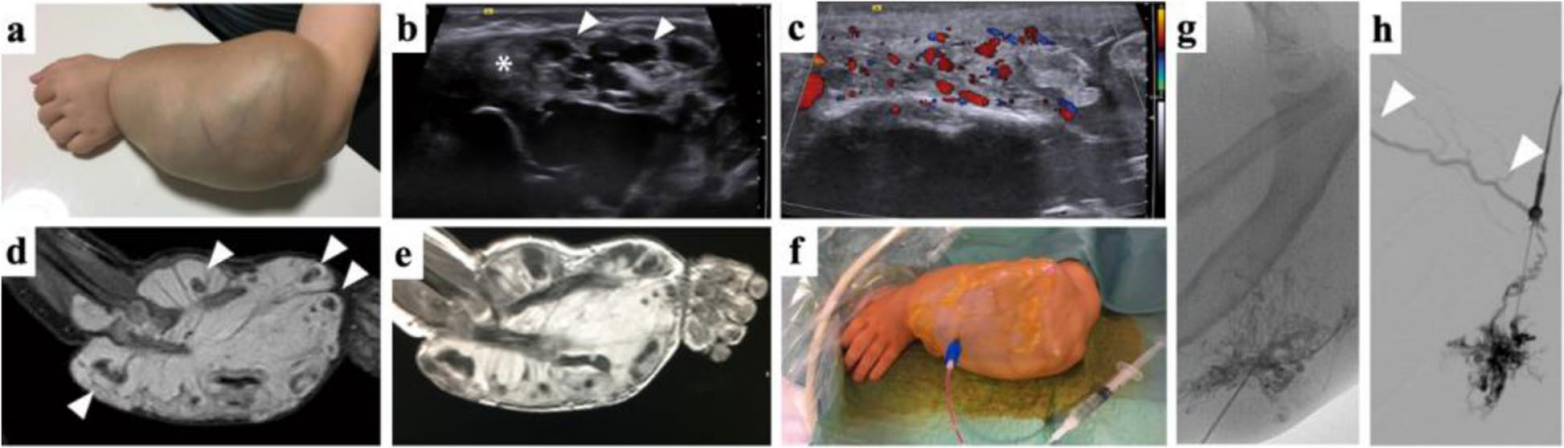
Venous Malformation a four-year-old child of the forearm with bone disfigurement. **a** Clinical presentation shows large, non-pulsatile painless soft tissue mass with bluish discoloration and visible dysplastic veins. Wrist function was restricted but finger motility was not significantly impaired. The heavy weight of the arm made the boy carry his left arm with the right hand. **b** B-Mode ultrasound imaging shows cavernous dysplastic veins (white arrowhead) and thrombosed parts (white asterisks) of the lesion. **c** Color-coded Duplex ultrasound reveals slow blood flow within the dysplastic vessels. **d** STIR MRI imaging shows typical hyperintense signal with multiple hypointense spots representing phleboliths (white arrowheads). T1-weighted MRI imaging after contrast administration shows enhancement of the large venous cavities of the VM (**e**). Venous malformations are treated by percutaneous sclerotherapy. The periprocedural image (**f**) shows confirmation of blood aspiration after percutaneous access to the dysplastic vessels, before using a contrast agent and a sclerosing agent subsequently. **g** Angiographic depiction of VM after contrast agent administration via percutaneous access displays a fine reticular network of dysplastic vessels and drainage to deep veins (**h**) not visible on MRI

*VMs* as the most common type of simple vascular malformations occur with a prevalence of 1–4% in the population [52]. They appear mainly sporadic. Common VMs present as focal, multifocal, or diffuse manifestations, as well as part of syndromic disorders such as Blue rubber bleb nevus (Bean) syndrome [53]. Errors in the development of the venous network are leading to dilatated and dysfunctional, irregular, and thin-walled veins and venules with scant, often asymmetric smooth muscle cells, which can involve any organ and multiple tissue types. VMs manifest characteristically as soft, non-pulsatile, and compressible lesions with bluish discoloration of the skin that increase in volume with dependent position, extremes of temperature, or Valsalva (Fig. 2) [54]. Pediatric VMs may be stimulated to grow by hormonal changes at puberty as well as following trauma or partial resection [55, 56]. Though VMs are present at birth, they may clinically manifest later in life, particularly in case of deep lesions as intramuscular malformations. Superficial lesions usually show a bluish/purplish discoloration and an association with dilated veins [57]. Symptoms of VMs include pain, swelling, disfigurement, functional impairment, and localized coagulopathy with raised fibrin degradation products, which may cause recurrent thrombosis and thrombophlebitis. The latter, a unique chronic consumptive coagulopathy, appears especially in large lesions whereas the ongoing consumption of clotting factors owing to stasis in the VM can result in severe systemic coagulopathy in children accompanied by low fibrinogen levels, low factor XIII, and increased D-dimers. This can significantly impair the daily activities of a child. Furthermore, the negative impact on the quality of life consecutively contributes to a family’s decision to pursue rather aggressive treatment strategies [58, 59].

In addition to confirmatory diagnosis, imaging in VMs is intended to answer specific questions for treatment planning. Exemplarily, it can be determined whether the lesion is predominantly characterized by accessible caverns or by thrombosed portions inappropriate for puncturing. Furthermore, the question of rapid venous drainage into the deep venous system can be addressed. Regarding this, the currently most established imaging modalities providing detailed information are ultrasound and MRI.

On US, venous malformations appear as hypoechogenic, barely thrombosed hyperechogenic clusters of dysplastic nodular or tubular veins with heterogenous echotexture. Phleboliths present as hyperechogenic spots with posterior acoustic shadowing. Whereas focal superficial lesions are well defined and compressible, diffuse VMs show various varicosities penetrating numerous tissue planes (Fig. 2). The exact definition of the extent of a VM can be challenging, as slit-like protrusions of the lesion tend to spread along connective tissue spaces and may be disguised by the limited contrast-resolution power [16]. CDUS demonstrates typical monophasic venous flow, very slow or no-flow pattern [60]. In addition, the presence, patency, and valve function of the deep and superficial venous system, can also be evaluated using ultrasound. This is particularly important in the pre-treatment assessment of VMs. It could be demonstrated recently, that color-coded CEUS with integrated perfusion analysis allows for differentiation between healthy tissue, untreated VM, and with gelified ethanol–treated tissue and hereby might assist in therapy guidance and monitoring [19].

MRI is considered the imaging modality of choice especially in cases of deeper VMs and their precise determination of tissue involvement (subcutaneous tissue, skin, muscles, bone, tendons). In the use of MRI, some prognostic questions can be answered by demonstration of tissue or joint involvement and the evidence of prior hemorrhage. Exemplarily, there is a predictive risk of post-treatment contractures in relation to the degree of muscle involvement of VMs [61, 62]. Compared to the surrounding fat tissue, VMs appear characteristically hyperintense on T2-weighted (T2w) images and fluid-sensitive sequences (e.g., STIR) as well as hypointense or isointense to muscle on T1-weighted (T1w) images. If high T1 signal intensity is discovered within the malformation, this may be associated with intralesional thrombosis, hemorrhage, or fat. In comparison to LMs, fluid–fluid levels are less common. Phleboliths present as scattered rounded areas of low signal intensity in all pulse sequences. The absence of flow voids clearly distinguishes VMs from AVMs. On contrast-enhanced MRA, VMs demonstrate early venous enhancement and slow gradual filling with contrast on delayed venous imaging, whereas there is an absence of arterial enhancement. On delayed postcontrast T1w images diffuse enhancement of the venous channels is typical for VMs. In addition, delayed postcontrast images are also useful in treatment planning by identifying any connection between VMs and the deep venous system increasing the potential risk of thrombosis and decreasing the effectivity of sclerotherapy in children. Therefore, the categorization of VMs according to the Puig classification plays a crucial role [63]: Type I VMs present isolated without detectable drainage into surrounding veins, type II VMs drain into non-dilated normal veins while type III VMs reveal drainage into dilated and type IV VMs into dysplastic veins. In the last two categories, minimally invasive treatment has to be monitored carefully due to the risk of agent dislocation in draining veins with systemic thromboembolism.

Using direct percutaneous phlebography, VMs present as clusters of dysplastic veins with contrast pooling without any signs of relevant arterio-venous shunting [13]. Due to the informative superiority, the non-invasive nature, and the lack of ionizing radiation of the available imaging alternatives, phlebography is not the first diagnostic modality of choice in a child pre-procedural, but can provide additional information in location, extension, and patency of the lesion during the procedure as well as its drainage to deep veins.

Phleboliths which are pathognomonic for VMs can be detected both on cross-sectional imaging as well as on conventional x-rays [64].

#### Lymphatic malformations

**Fig. 3:**
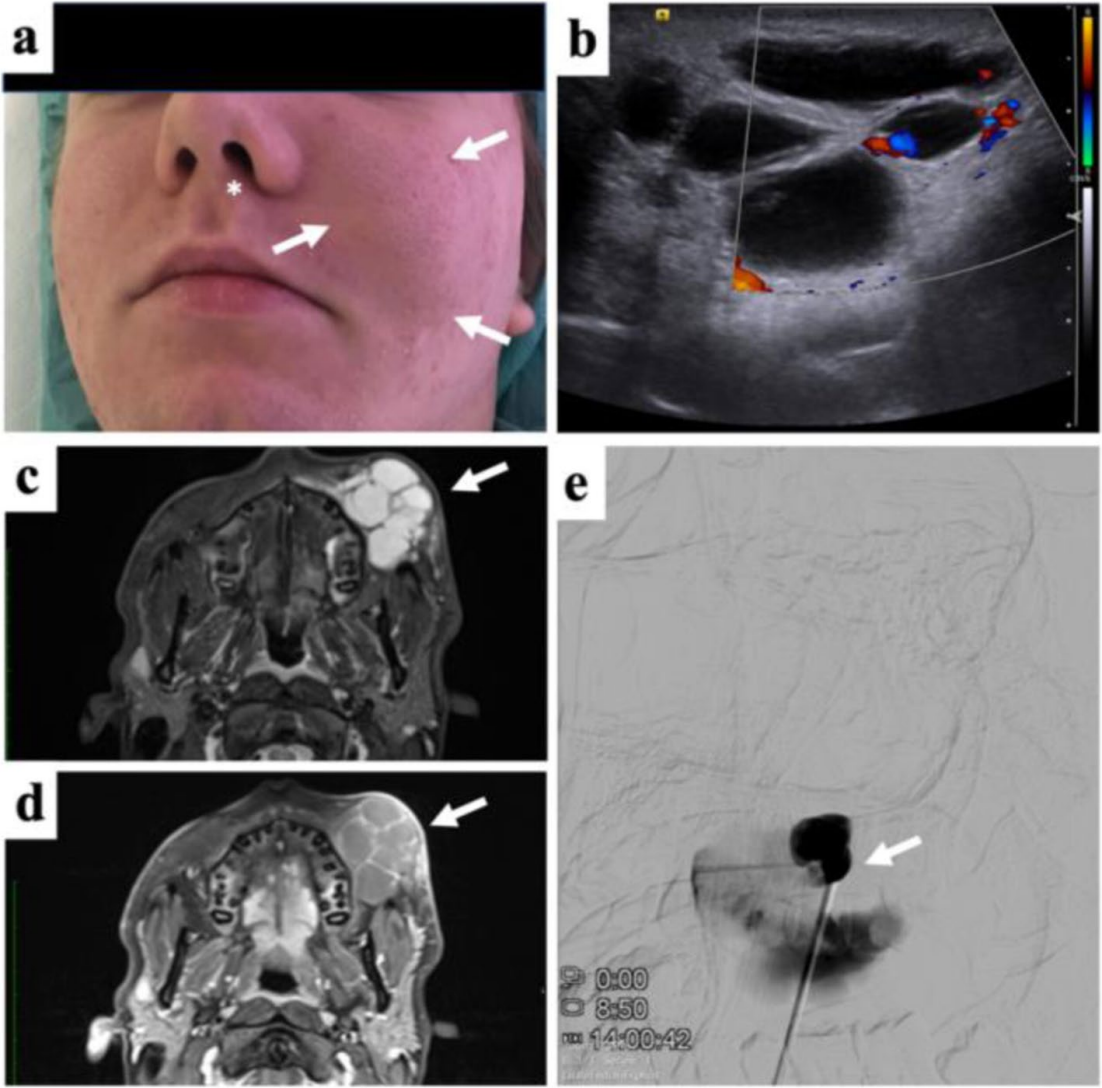
Subcutaneous Lymphatic malformation of the face in an adolescent. **a** Clinical photograph showing local swelling and moderate discoloration of the cheek (white arrows) and local deviation of the left ala of the nose (asterisk). The patient was suffered from esthetic disfigurement with the mass itself being painless. **b** Color-coded Duplex ultrasound documents the absence of intralesional flow, only focal vascularization along the septa. **c** Fat-saturated STIR MRI shows fluid-filled cavities in the subcutaneous tissue of the mixed-type lymphatic malformation (arrow). **d** Following contrast administration only septal structures show moderate enhancement (arrow). **e** Percutaneous contrast administration before sclerotherapy reveals partial communication between adjacent lymphatic cysts. Sclerotherapy resulted in subtotal regression of the mass

LMs, similarly common as CMs while appearing with a prevalence of 0.1–2% in the population, consist of dilated lymphatic channels and spaces without connection to the lymphatic system. While classified as microcystic (e.g. cyst size < 2 cm), macrocystic (e.g. cyst size ≥2 cm), and mixed type, these lesions can be present at birth or appear later [65]. It has to be said that there is no clear consensus regarding the cyst size differentiating micro- and macrocystic LM. LMs mostly affect the cervicofacial and axillary areas while potentially occurring at any area of a child’s body as a flesh-colored soft mass (Fig. 3). Characterized by the occurrence in early childhood, generally, they are regularly treated timely before transition to adult practice. They can enlarge fast and be blue colored in case of intermittent infections or internal hemorrhage. Functional impairment of nearby structures may result in pain and swelling whereas disfigurement of affected areas and cellulitis is demonstrated frequently. In addition, microcystic lesions may be accompanied by localized fat hypertrophy, which is essential to recognize as this is a limiting factor for the amount of bulk reduction, and the children’s and parents’ expectations should be handled accordingly. Generalized LMs can present as generalized lymphatic anomaly (GLA) and as Gorham–Stout disease (GSD). While the latter mainly aggressively involves the bones, GLA is characterized by multifocal cystic lesions affecting numerous organ systems as well as the bones. Additional clinical presentations in children suffering generalized LMs include hepatic cysts, splenic cysts, and pleural effusions [66, 67]. To date, the established imaging modalities for LMs are US and MRI.

In US, LMs appear as septate or loculated hypoechoic/anechoic, no-flow lesions [68]. Macrocystic lesions characteristically present multiple, anechoic cystic spaces with thin internal septations in which there may be seen varying degrees of floating echos in case of hemorrhage or infection. Microcystic lesions demonstrate an ill-defined echogenic mass with scant minute cysts, or no cysts visible at all. CDUS regularly shows no or minimal flow within both the micro- and macrocystic lesions [60].

MRI may define the complete extent of the deep-situated LMs more accurately. The typical appearance of macrocystic LMs is a lobulated, multiloculated mass showing low to intermediate signal intensity on T1w images as well as high signal intensity on T2w images. Fluid-fluid levels may be frequent. Faint capsular and septal enhancement sparing the cystic cavities themselves are notable after administration of contrast medium. Though, in case of a dense cluster of microcysts, the lesion may reveal diffuse enhancement. If there is any concomitant inflammation or infection of the LM, enhancement of the adjacent soft tissue or surrounding edema may be seen [30, 48].

CT, which may be used as a primary diagnostic tool only in case of contraindications to MRI, displays homogenous cystic lesions whereas there may be also heterogenous densities reflecting hemorrhagic or proteinaceous content [60]. However, similar to VMs, CT should be avoided due to associated radiation burden whenever possible.

#### Fast-flow malformations

##### Arteriovenous malformations and arteriovenous fistulas

AVMs and AVFs as fast-flow lesions represent less than one-third of vascular malformations. Both may be sporadic or associated with syndromes such as hereditary hemorrhagic telangiectasia (HHT), CM-AVM, or Parkes Weber syndrome [65]. They are results of an abnormal direct connection between arteries and veins without an intermittent capillary bed, which persist since embryonic development. In AVFs, single or a small number of direct connections exist between arteries and veins, whereas in AVMs, there is a low-resistance net-like nidus linked to multiple feeding arteries and draining veins. The normally interposed capillary bed is either partly or entirely lacking [69]. Histologically characterized by thick-walled arteries and arterialized thick-walled veins, fast-flow malformations most commonly appear in the head and neck region. Of note, intracranial AVMs are more frequent than extracranial AVMs. Peripheral AVMs are habitually located in the limbs, trunk, and viscera [70]. On clinical examination, superficial lesions may present slightly compressible with pink-red cutaneous stain, warmth, and a palpable bruit or thrill. Due to the fast-flow shunting, there is no gradual pressure decrease from the arterial pressure to the low pressure on the venous side. This may have consecutive hemodynamic effects resulting in peripheral ischemia, local hypervascularity, steal phenomenon, and increased venous pressure. Thus, the children may present with possible clinical features as pain, bleeding, hyperemia, tissue overgrowth, ulceration, and gangrene up to high-output cardiac failure [71, 72]. According to the Schobinger classification, the natural history is comprised of four distinct stages: quiescent, growing, symptomatic, and decompensating [73]. Like AVMs in adults, therapy decisions can present an absolute challenge. During a lifetime, AVMs tend to constantly grow. Part of the childhood lesions may show episodes of rapid progress, characteristically around puberty. Though, it is challenging to predict which AVM will behave like this further complicating adequate treatment decisions. A significant long-term reaction of a pediatric AVM located in direct proximity to a growth plate is due to its impact on bone growth. Regarding these lesions, therapy should be conducted even if the children present symptom free [74].

**Fig. 4:**
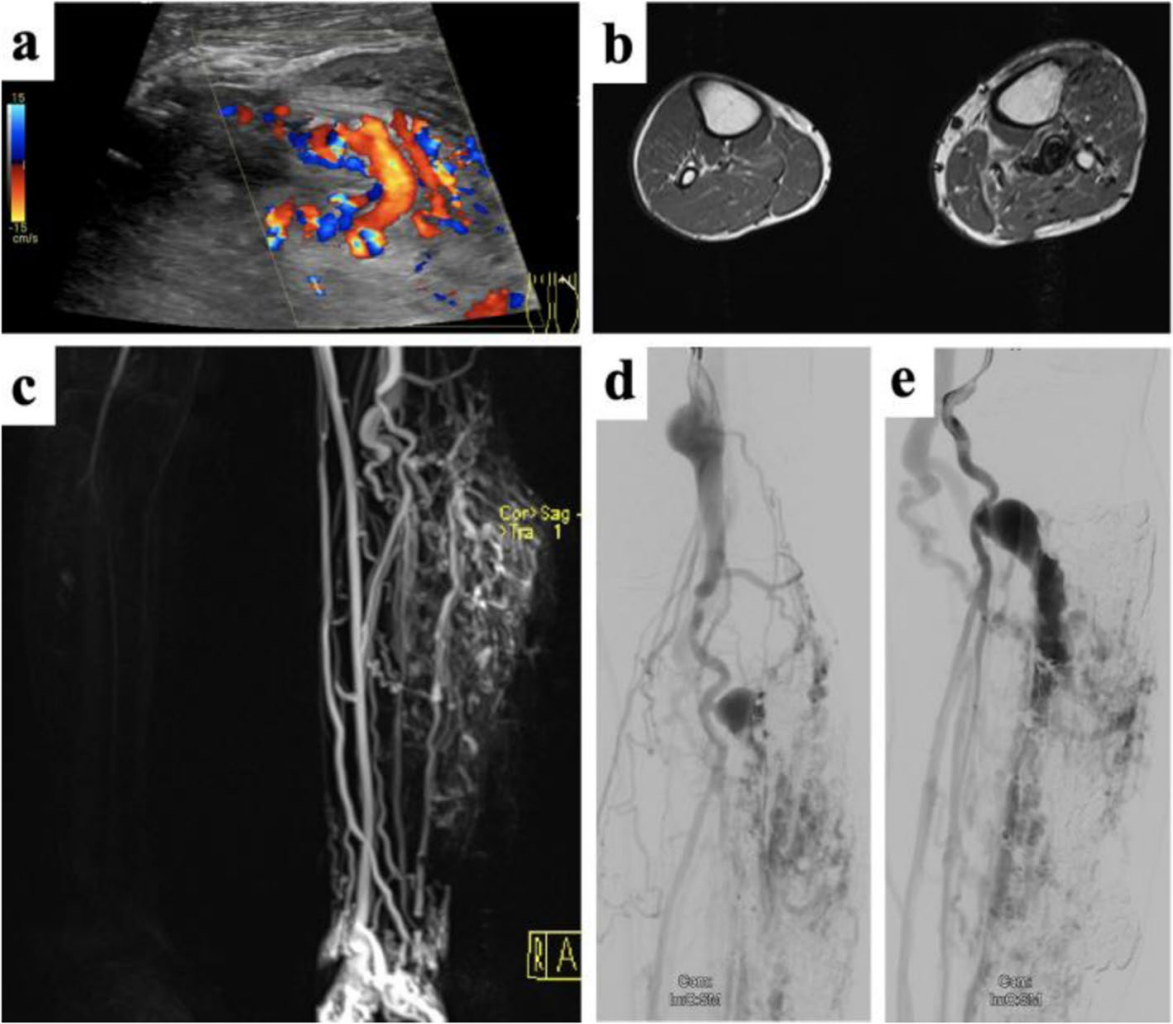
Arteriovenous malformation of the lower limb with associated soft-tissue proliferation. **a** Color-coded Duplex ultrasound reveals vigorous flow along the AVM nidus. **b** Cross-sectional MRI reveals multiple flow-voids as sign of arterialized flow patterns together with soft-tissue proliferation adjacent to the AVM. The mass was clinically pulsatile without ulceration at the time of clinical presentation. **c** Dynamic MR angiography shows rapid filling of the AVM along the left lower limb at time with only initial enhancement of the arteries of the non-affected leg. Pre-embolization angiogram in the late arterial phase (**d**) and early venous phase (**e**) depicting dilated aneurysms along the venous outflow tract

Imaging has a key role in diagnostics and, in particular, in treatment planning of AVMs, e.g., by revealing whether the transarterial access route may be sufficient by itself or if a transvenous and/or percutaneous direct puncture approach should be chosen instead or additionally. To confirm the diagnosis, it is important to identify the fast-flow patterns, for which both ultrasound and MRI are well established (Fig. 4).

On US, AVMs appear as irregular conglomerates of enlarged and tortuous vascular channels with dilated draining veins, combined with sparse echoic vascular stroma. Owing to the mechanical impact of fast-flow malformations, the surrounding tissues may also present hyperechoic or thickened [17]. There may be a difficult delineation to the surrounding tissue caused by convolutes of very thin vessels, which can appear rather homogeneous in B-mode (pseudo-soft tissue) but frequently impressive in CDUS. Using the latter, the lesion generally presents with nearly complete color filling and multidirectional, pulsatile flow within the lesion and vibration artifacts in the tissue surrounding the fistula/nidus (color bruit artifact, confetti sign). SWA shows spectral broadening and low resistance in the supplying artery, turbulent flow in the connecting channel, arterialized pulsatile flow in the draining vein [17, 60]. CEUS can display a fast crossing of microbubbles from the arterial to the venous structures in extensive high-flow malformations, sometimes demonstrating multiple shunts when using a high-resolution technique [75].

Flow voids in MRI are pathognomonic for fast-flow lesions. MRA and dynamic sequences display the hemodynamic pattern of the lesion with early venous filling and allow to identify the feeding arteries and draining veins [32, 76]. Compared to other vascular anomalies which may be considered for differential diagnosis, AVMs are not composed of a well-defined, solid mass on MRI. Perilesional soft tissue changes and contrast enhancement may appear similar to discrete tissue masses, exemplarily if the malformation is limited by a muscular sheath, surrounded by edema, or causing fatty hypertrophy, which makes the diagnosis challenging [77]. This distinction may be even more complicated in the presence of syndromic diseases; AVMs in patients presenting a mutation in *PTEN* (phosphatase and tensin homolog) gene are characterized by an association with variable amounts of hyperplastic ectopic fat at the same site as the VA, which may mimic a round vascular mass [78]. Time-of-flight sequences and four-dimensional-flow MRA are used to evaluate intracranial AVMs, though cerebral CT-angiography (CTA) remains the most established modality in the assessment of these lesions [79].

Regarding peripheral AVMs, CTA can show enlarged and tortuous supplying arteries with early venous filling owing to fast-flow shunting. However, due to the less diagnostic information compared with MRA and the high radiation exposure, CTA does not represent an adequate choice for diagnostic purposes, particularly in the young VA patient population. An exceptional aspect of this is treatment planning in complex and extensive AVMs. Here, time-resolved 4D CTA was recently suggested to better depict the numerous supplying arteries to the nidus and multiple dominant draining veins [80]. In the use of a wide-ranging scan field, perfusion dynamics of the entire affected limb can be evaluated. As perfusion imaging requires repetitive scanning over a certain time period, the radiation burden has to be critically weighted against the potential benefits [13, 81, 82]. In case of further specific situations, such as if the AVM involves bone, CT may be of particular interest in the assessment of the surrounding tissues [64].

Although it can be useful in diagnosis by full depiction of arteriovenous shunting, nidus, and venous outflow, catheter angiography via transarterial, transvenous, or percutaneously direct puncture, is an invasive procedure, that should be reserved for periprocedural treatment planning.

## Drawbacks of current imaging concepts

The current, well-established imaging concepts, including B-mode US, CDUS, CEUS, 4D CTA, MRI, dynamic MRA, and periprocedural arterial and venous angiographic imaging, provide detailed information on the morphological characteristics and the extent of the malformation, including hemodynamics [83]. Several of these modalities, exemplarily MRA, that relies on first-pass or equilibrium contrast material enhancement, additionally enable to study the microvascular circulation of vascular malformations. Here, physiologic parameters that can be extracted are flow, perfusion, vascular volume fraction, permeability, and/or the vascular wall structure. However, current modalities also present drawbacks for diagnostic vascular malformation imaging as there are still technical limitations in imaging the microvascular aspects of the disease. However, some important characteristics in particular regarding AVMs remain concealed for current imaging techniques: the specific assessment of local perfusion pressures and the distinct manifestations of steal effects, can only be guessed at based on the currently used modalities and currently cannot be deduced to patients’ symptoms or future course of disease.

In addition, molecular aspects are becoming increasingly important as the understanding of the pathogenesis and progression of vascular malformations is improving: This knowledge includes the fundamental pathological and histological phenotypes of a variety of cell types in different subtypes of vascular malformation and the molecular profiles of the affected vasculatures enabling the use of anti-angiogenic or anti-proliferative drugs, derived from oncology [84, 85]. Current imaging modalities fail to report the biological activity of the vascular malformation. For this purpose, novel imaging techniques targeting specific molecular markers may be the most prolific approach. Consequently, there is an increasing demand for imaging enabling visualization of angiogenesis to provide quantitative outcome measures which may be used to predict the response to targeted therapy and the risk of recurrence as well as to monitor the follow-up of treatment efficacy [86]. Using a single technological platform including anatomical, functional, and (patho-)genetic information for diagnosis and therapy (theranostics) of VA is broadly desired.

## Novel imaging concepts and future perspectives

Molecular imaging can be generally distinct as the in vivo measurement and characterization of biologic processes at the cellular and molecular level. Thereby, in contrast to “classical” diagnostic imaging, it targets to examine the molecular alterations which are forming the basis of the related disease rather than to image the morphological end effects of these molecular abnormalities [87].

Regarding the molecular pathogenesis of vascular malformations, the major pathways are vascular endothelial growth factor (VEGF), Ras/Raf/MEK/ERK, angiopoietin-TIE2, transforming growth factor beta (TGF-β), and PI3K/AKT/mTOR, which are involved in controlling cellular growth, apoptosis, proliferation, differentiation, and play an essential role in endothelial cell signaling and angiogenesis [88–90]. According to the pathway, the uncontrolled linking of specific key agonists or endogenous inhibitors of angiogenesis to their corresponding target receptors expressed on the activated endothelial cells lead to pathogenic pro-angiogenic stimuli [88]. In the use of molecular imaging, it is possible to target specific imaging probes to these markers on the endothelial surface of vascular malformations [87]. We review initial results from pioneering imaging studies which have targeted specific biomarkers of vascular malformations and attested to the feasibility of the method.

### Radionuclide imaging

**Fig. 5:**
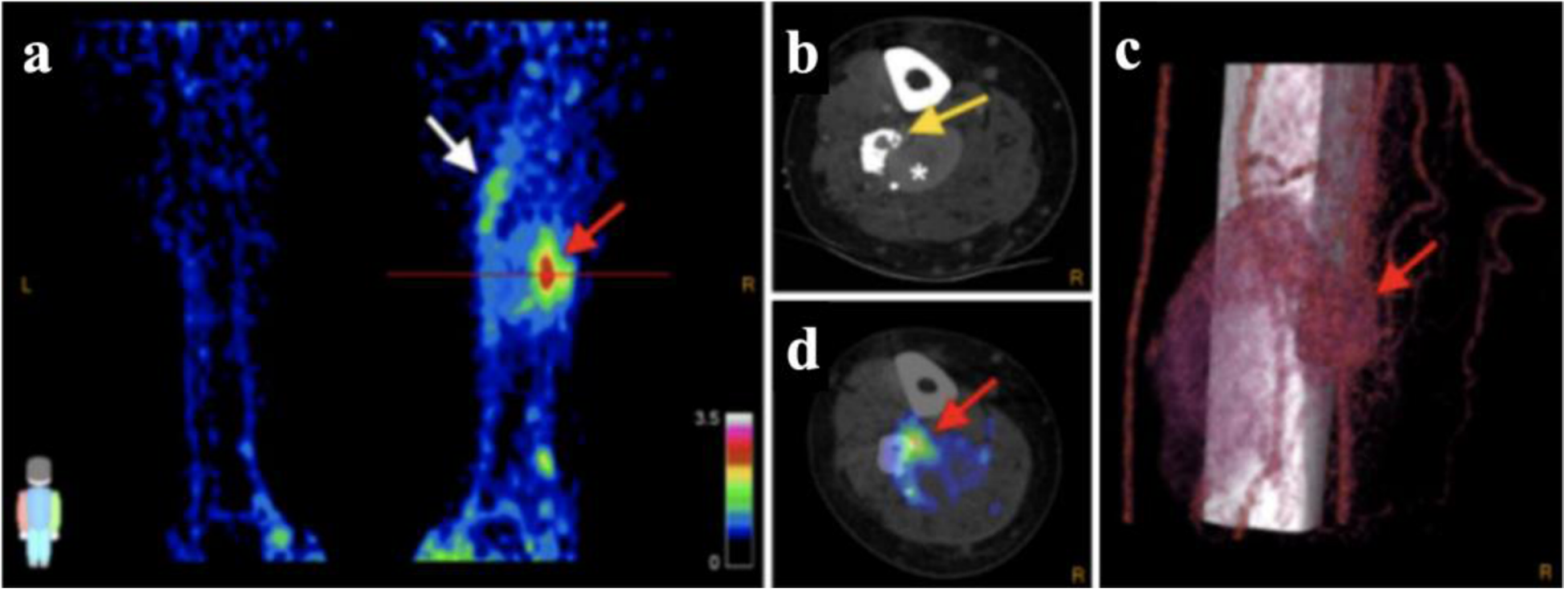
^68^Ga-RGB PET/CT in 38-year-old man with right-leg AVM. Maximal-intensity ^68^Ga-RGD PET projection (**a**), CTA (**b**) and 68Ga-RGD PET/CT (**d**), and 3D reconstruction of 4D-CTA (**c**) show nidus (SUV_ma*x*_ 3.2; SUV_peak_ 2.5, red arrows). Axial plane in (**b**) and (**d**) is at location of line in (**a**). Heterogeneous pattern of enhanced uptake is seen in tissue adjacent to nidus at more proximal part of the right leg (white arrow); bone deformation in the fibula caused by compression and infiltration of vessels is also seen (yellow arrow), along with large venous aneurysm (asterisk). Arterial flow with nidus and fistula is seen in (**c**), along with upcoming venous flow and the large venous aneurysm. This research was originally published in JNM. Lobeek D, Bouwman FCM, Aarntzen EHJG, Molkenboer-Kuenen JDM, Flucke UE, Nguyen HL, Vikkula M, Boon LM, Klein W, Laverman P, Oyen WJG, Boerman OC, Terry SYA, Schultze Kool LJ, Rijpkema M. A Clinical Feasibility Study to Image Angiogenesis in Patients with Arteriovenous Malformations Using ^68^Ga-RGD PET/CT. J Nucl Med 2020; 61:270–275.© SNMMI

Radiolabeled arginine-glycine-aspartate tripeptide sequence derivatives (RGD) and positron-emission tomography/computed tomography (PET/CT) allow quantitative molecular imaging of angiogenesis in the use of the adhesion molecule integrin αvβ3 as the target marker for radiopharmaceuticals, expressed on the endothelial surface of newly formed vessels [91–93]. Whereas there was lacking expression in other vascular malformations such as cavernous malformations, integrin αvβ3 expression could be initially shown within cerebral AVMs [94]. Gallium-68 labeled dimeric RGD (^68^Ga-RGD) is binding integrin αvβ3 with high affinity, as manifold demonstrated in tumor imaging [95–97]. A recently published [86] pilot study aiming to image proliferative angiogenesis in patients with AVMs using ^68^Ga-RGD PET/CT showed increased ^68^Ga-RGD uptake with good contrast to noise ratio in the nidus of peripheral AVMs, validated by immunohistochemical analysis confirming cytoplasmatic and cell membranous expression of αvβ3 integrins in endothelial cells (Fig. 5). Consequently, this enabled the delineation between intralesional regions with and without enhanced integrin αvβ3 expression within an AVM. Furthermore, auspicious findings were additional detected foci of integrin αvβ3 expression in comparison to conventional angiographic imaging and a well-defined nidus in a complex AVM which was not identified on “conventional” imaging. Thereby, this novel imaging approach has the potential to identify treatment targets of active AVM parts, especially in patients showing no clear nidus on conventional imaging as well as in patients with large size or complex AVMs [86]. However, it will need further studies whether ^68^Ga-RGD PET/CT or prospectively PET/MRI could be used to assess the angiogenic “activity” of AVMs, which may also be of prognostic value.

### Photoacoustic imaging

**Fig. 6:**
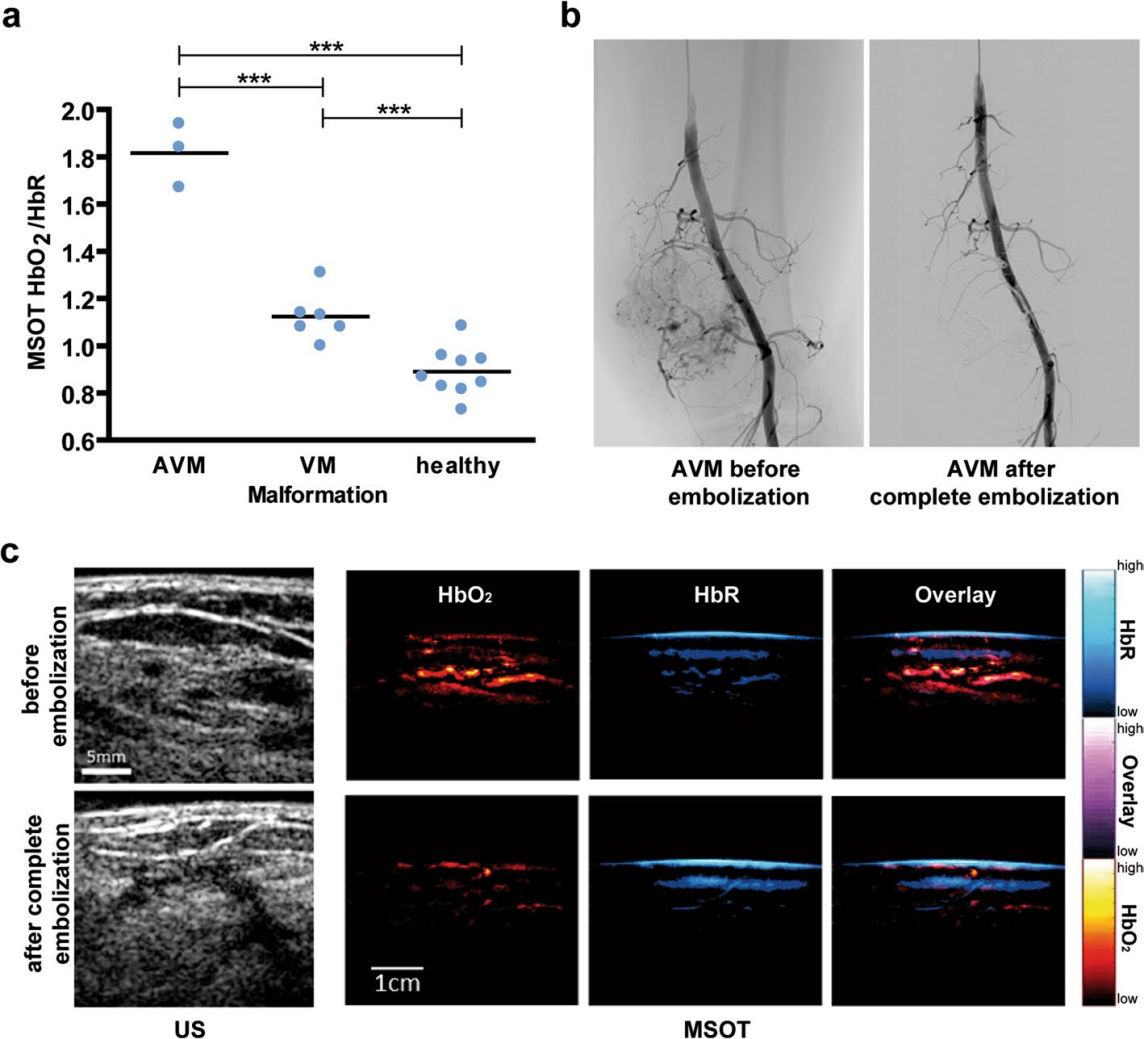
Multispectral optoacoustic tomography (MSOT) of vascular malformations. **a** Novel hybrid MSOT/US technique was shown to enable distinction of arteriovenous (AVM) from venous (VM) malformations as well as from healthy tissue by non-invasive, contrast-free, and radiation-free imaging of the lesions at the patients’ bedside. Further, as exemplarily shown for a patient receiving complete embolization of an AVM (**b**), MSOT/US depicted therapy response by showing a reduced signal of oxygenated and deoxygenated hemoglobin (**c**). ***p < 0.001. Figure adapted from Masthoff M, Helfen A, Claussen J, Karlas A, Markwardt NA, Ntziachristos V, Eisenblätter M, Wildgruber M. Use of Multispectral Optoacoustic Tomography to Diagnose Vascular Malformations. JAMA Dermatol. 2018 Dec 1;154(12):1457–1462. doi: 10.1001/jamadermatol.2018.3269. PMID: 30267083; PMCID: PMC6583374© 2018, American Medical Association

Besides radionuclide imaging, the presented imaging methods to diagnose and monitor VAs all reflect anatomical morphological characteristics of these lesions. Radionuclide imaging on the other hand requires radioactive tracers, which limits its broad application in the mostly young cohort of VA patients. Further, as stated above, MRI is elaborate in very young patients requiring general anesthesia or deep sedation and may come along with the need for repetitive contrast administration during the course of disease and treatment monitoring. Therefore, an imaging technique that addresses functional biomarkers of VA which at the same time is radiation free, easy to use, and contrast free would be desirable. In this context, multispectral optoacoustic imaging (MSOT) is an emerging and promising technique, which is based on the photoacoustic effect [98, 99]. Briefly, pulsed laser excitation at multiple wavelengths causes thermoelastic expansion of the scanned tissue, which thereby transmits ultrasound waves that can be used for image generation. Due to specific properties of intrinsic absorbers, the spectral signal can be used unmixed and semiquantitative data of intrinsic tissue biomarkers such as oxygenated and deoxygenated hemoglobin, lipids, water, or melanin can be calculated. Considering the low anatomical resolution of MSOT, the technique has been applied in the clinical context mainly using a hybrid approach combining MSOT and US imaging [100–103]. MSOT/US has been shown to be able not only to display healthy human vasculature but also to distinguish between fast-flow and slow-flow vascular malformations [104]. In a pilot study, the hybrid approach also enabled to monitor the therapy effects of endovascular embolization in AVMs as well as of percutaneous sclerotherapy in VMs, demonstrating the potential for its application for therapy monitoring in the future (Fig. 6). Moreover, optoacoustic imaging has been used to identify lymphatic vessels and to guide lymphatic microsurgery using indocyanine-green as the contrast agent [105]. Hence, an application of MSOT for lymphatic malformations does not seem too far-fetched, potentially even without the need for contrast agents utilizing the high lipid content of lymph and chylus. However, next to simultaneously imaging anatomical and functional characteristics of the dysplastic vessels themselves, MSOT/US might also be of value to asses functional effects of AVMs to tissue distant to the lesion (e.g., steal effect due to high shunt volume with consecutive ischemia) since the technique was shown to be able to identify undersupplied tissue within cuff-induced ischemia as well as in peripheral arterial disease patients [106]. Further, high-resolution photoacoustic imaging by the means of raster-scanning optoacoustic mesoscopy (RSOM) might be also of interest for imaging of VA’s with skin involvement in the future, since the technique enables to analyze the microvasculature and its hemodynamics [107] as well as to display effects of vascular targeted therapies [108], which may become even more relevant in future treatment algorithms considering the new insights in genetic and molecular pathways in VA development and progression [109]. But photoacoustic imaging is still limited regarding penetration depth (up to 3–4 cm) and further evaluation of the technique’s value for clinical routine has to be shown in larger prospective studies.

### Thermography

**Fig. 7:**
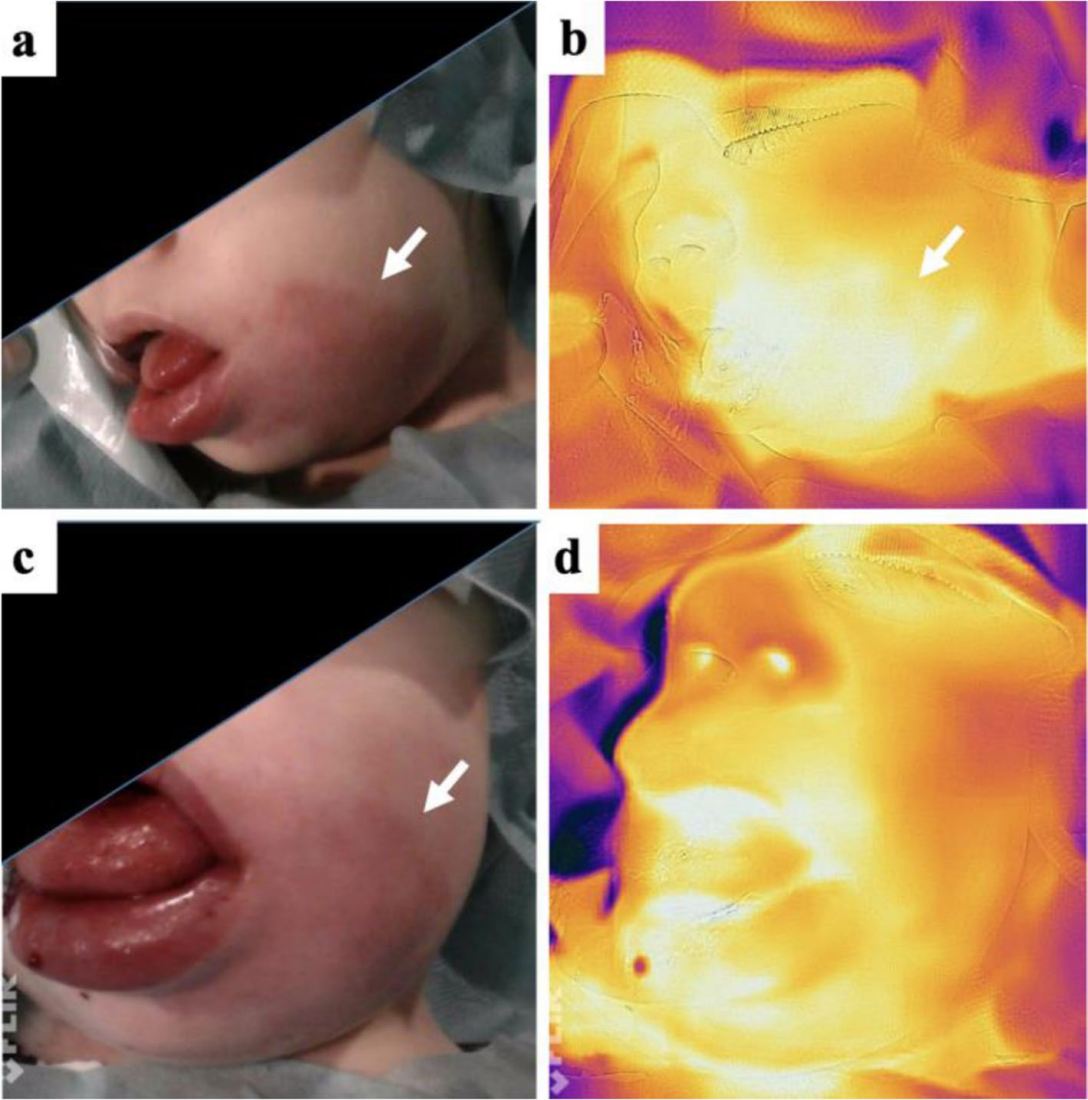
Infrared thermography of an arteriovenous malformation of the face. **a** The patient (m, age: 3 years) suffers from an AVM of the left cheek and lower lip. Pink discoloration of the skin is characteristic of the arterial component in this vascular malformation. **b** Infrared thermography before embolization. Note the locally increased temperature, color-coded as white areas. **c** Clinical presentation and **d** infrared thermography immediately after successful embolization. The locally increased temperature is gone. Note a local swelling, induced by the embolization disappearing after a few weeks. AVM is marked with arrow, a more whitish color indicating elevated temperature

Infrared thermography is a non-invasive imaging technique to detect infrared electromagnetic radiation, thereby temperature changes are being measured (104, 105). In humans, measurable skin temperature changes are mainly produced by superficial blood flow alterations. Hence, it is a suitable tool to measure perfusion changes in superficial vascular malformations, e.g., before and after intervention or during follow-up. Especially fast-flow vascular malformations are warmer than the surrounding skin due to a locally increased perfusion in the involved area. In case of AVMs, perfusion and thus temperature is increased close to the lesion and superficial draining veins (Fig. 7) and may be reduced downstream in case of vascular steal. After intervention perfusion may initially be increased (inflammatory reaction) and normalized during follow-up. Quantification of the infrared thermograms may be performed using specific software (106). Supplementary, in patients that can not specify their pain level, pain scale can be measured by thermography: in regions with high pain level, parasympathicus is activated, and therefore, perfusion and temperature are decreased. Thermography is used so far for objective evaluation and follow-up on infantile hemangiomas (107) and AVMs (108). In contrary to fast-flow lesions, a superficial slow-flow lymphatic or venous malformation in the absence of inflammation does not show any temperature changes in comparison to the surrounding tissue.

## Conclusions

In today’s clinical routine, vascular malformations are diagnosed by using US and MRI complementary to detailed clinical examination. As misdiagnosis and false classification of vascular malformations are frequent, imaging modalities easy to use and to interpret are of utmost importance. In addition, the recently improved understanding of pathogenesis and progression forces visualization of the underlying biological activity and angiogenesis in vivo. Thus, novel techniques for diagnosis, patient/treatment selection, and treatment monitoring are required to improve patient outcome. The attractiveness of imaging and treating the same molecular target is appealing. A few pioneering imaging studies including PET and MSOT have successfully targeted highly specific aspects of vascular malformations while attesting to the feasibility of such methods.

## Data Availability

Not applicable.

## Abbreviations

AVF: Arteriovenous fistula
AVM: Arteriovenous malformation
B-mode: Brightness mode
CDUS: Color-coded Duplex ultrasound
CEUS: Contrast-enhanced ultrasound
CM: Capillary malformation
CM-AVM: Capillary malformation–arteriovenous malformation
CMTC: Cutis marmorata telangiectatica congenita
CTA: Computed tomography angiography
DCE-MRA: Dynamic contrast-enhanced magnet resonance angiography
DSA: Digital subtraction angiography
FSE: Fast spin echo
Ga: Gallium
GLA: generalized lymphatic anomaly
GRE: Gradient-echo
HHT: Hereditary hemorrhagic teleangiectasia
HR-US: High-resolution ultrasound
ISSVA: International Society for the Study of Vascular Anomalies
KTS: Klippel–Trenaunay syndrome
LM: Lymphatic malformation
LSCI: Laser speckle contrast imaging
MRI: Magnetic resonance imaging
MRA: Magnetic resonance angiography
MRV: Magnetic resonance venography
MSOT: Multispectral optoacoustic tomography
PC: Phase contrast
PDI: Pulsed dye laser
PRF: Pulse repetition frequency
PW-Doppler: Pulsed wave Doppler
RCM: Reticulated capillary malformation
RGD: Radiolabeled arginine-glycine-aspartate tripeptide sequence derivatives
RSOM: Raster-scanning optoacoustic mesoscopy
SE: Spin echo
SI: Signal intensity
STIR: Short tau inversion recovery
SWA: Spectral waveform analysis
SWS: Sturge–Weber syndrome
T1w: T1-weighted
T2w: T2-weighted
TGF-β: Transforming growth factor beta
TOF: Time-of-flight
US: Ultrasound
VA: Vascular anomaly
VEGF: Vascular endothelial growth factor
VM: Venous malformation
